# Low muscle mass assessed by psoas muscle area is associated with clinical adverse events in elderly patients with heart failure

**DOI:** 10.1371/journal.pone.0247140

**Published:** 2021-02-16

**Authors:** Takehiro Funamizu, Yuji Nagatomo, Mike Saji, Nobuo Iguchi, Hiroyuki Daida, Tsutomu Yoshikawa

**Affiliations:** 1 Department of Cardiology, Sakakibara Heart Institute, Tokyo, Japan; 2 Department of Cardiovascular Biology and Medicine, Juntendo University Graduate School of Medicine, Tokyo, Japan; 3 Department of Cardiology, National Defense Medical College, Tokorozawa, Japan; Maastricht University Medical Center, NETHERLANDS

## Abstract

**Background:**

Acute decompensated heart failure (ADHF) is a growing healthcare burden with increasing prevalence and comorbidities due to progressive aging society. Accumulating evidence suggest that low skeletal muscle mass has a negative impact on clinical outcome in elderly adult population. We sought to determine the significance of psoas muscle area as a novel index of low skeletal muscle mass in elderly patients with ADHF.

**Methods:**

In this single-center retrospective observational study, we reviewed consecutive 865 elderly participants (65 years or older) who were hospitalized for ADHF and 392 were available for analysis (79 years [74–85], 56% male). Cross-sectional areas of psoas muscle at the level of fourth lumbar vertebra were measured by computed tomography and normalized by the square of height to calculate psoas muscle index (PMI, cm^2^/m^2^).

**Results:**

Dividing the patients by the gender-specific quartile value (2.47 cm^2^/m^2^ for male and 1.68 cm^2^/m^2^ for female), we defined low PMI as the lowest gender-based quartile of PMI. Multiple linear regression analysis revealed female sex, body mass index (BMI), and E/e’, but not left ventricular ejection fraction, were independently associated with PMI. Kaplan-Meier analysis showed low PMI was associated with higher rate of composite endpoint of all-cause death and ADHF re-hospitalization (P = 0.033). Cox proportional hazard model analysis identified low PMI, but not BMI, was an independent predictor of the composite endpoint (Hazard ratio: 1.52 [1.06–2.16], P = 0.024).

**Conclusions:**

PMI predicted future clinical adverse events in elderly patients with ADHF. Further studies are needed to assess whether low skeletal muscle mass can be a potential therapeutic target to improve the outcome of ADHF.

## Introduction

Heart failure (HF) is a growing healthcare burden with increasing prevalence and comorbidities due to progressive aging society mainly in developed countries and a leading cause of hospitalizations and readmission [1]. As another issue in elderly population, sarcopenia emerged as a commonly seen, but undiagnosed, unappreciated condition. Sarcopenia is defined as a loss of muscle mass and function [2–4] and it has been increasingly recognized as an important risk factor for functional impairment, mental disorders, and poor quality of life [5–7] leading to the adverse health consequences such as disability, fall, fractures [8, 9] and increased mortality [10]. In advanced stages of chronic HF (CHF), a loss of skeletal muscle is commonly observed which contributes to impaired exercise capacity and frailty [11, 12], possibly initiating the vicious cycle ensuing with physical inactivity and malnutrition leading to further decline [13]. The prevalence of sarcopenia in patients with CHF was reported to be 20%, which was higher compared to healthy subjects [12] and a loss of muscle mass was associated with an unfavorable prognosis in patients with CHF [14]. However, sarcopenia has been assessed by various modalities and ununiformly defined cut-off values employed by many research groups [11, 12, 14], and the simple and reliable measures of sarcopenia have been limited so far. The psoas muscle area (PMA), which can be obtained from computed tomography (CT), has been shown to correlate well with whole body muscle mass [15] and can be potentially used as a surrogate marker. In recent studies, low PMA was associated with mortality and length of hospital stay in patients who underwent various types of intervention procedures, including transcatheter aortic valve implantation or cardiac operations [16, 17]. Few studies, however, have yet been conducted to examine the prognostic value of PMA in elderly patients with HF. The purpose of the present study was to investigate whether PMA as a novel index of skeletal muscle mass can predict clinical outcome in elderly patients with HF.

## Materials and methods

### Study design

The prospective HF registry is ongoing to collect data on the clinical characteristics and outcomes of patients who were hospitalized for acute decompensated HF (ADHF) at the Sakakibara Heart Institute. The clinical diagnosis of ADHF was made by the individual cardiologists according to the Framingham Criteria, specifically, the typical symptoms (orthopnea, breathlessness, nocturnal dyspnea, fatigue, tiredness), signs (elevated jugular venous pressure, hepatojugular reflux, third heart sound) evaluated by physical examination, and chest X-ray [18]. Patients presenting with acute coronary syndrome or isolated right-sided HF were excluded. Exclusive on-site auditing by the investigators (T.F. and Y.N.) ensured proper registration of each patient. In this retrospective observational study, we reviewed consecutive 865 elderly participants registered from January 2011 to December 2015. We defined elderly patients as being 65 years or older according to the World Health Organization definition. Patients were included if they underwent CT that spanned the L4 vertebrae during hospitalization or within one year before index hospitalization. The patients who did not undergo abdominal CT and those who lacked the value of height were excluded. Data analyses were completed using images collected by SOMATOM Definition Flash (Siemens Healthcare, Erlangen, Germany) or SOMATOM Sensation 16 (Siemens Healthcare, Erlangen, Germany) CT scanners. The cross-sectional areas of both sides of psoas muscles were measured using manual tracing at the bottom of the fourth lumbar vertebra (L4, S1 Fig). The mean value of both sides of PMA was normalized to the square of height to calculate the psoas muscle index (PMI). We defined low PMI as the lowest gender-based quartile of the PMI in accordance with the previous study [19]. The measurement of PMA was independently conducted by the physician who were blinded to the patient’s clinical characteristics. Clinical events such as death and ADHF rehospitalization after discharge were prospectively collected. The information on the cause of death (cardiac or non-cardiac) was also collected. The primary endpoint was defined as the composite of all-cause death and re-hospitalization for ADHF. This study was performed in accordance with the Declaration of Helsinki and approved by the institutional review board at Sakakibara Heart Institute and written informed consent was obtained from all study participants.

### Statistical analyses

In this study, we defined heart failure with preserved ejection fraction (HFpEF) as HF with left ventricular ejection fraction (LVEF) 50% or greater, heart failure with reduced ejection fraction (HFrEF) as HF with LVEF less than 40%, and heart failure with midrange ejection fraction (HFmrEF) as HF with LVEF between 40% and 49% [20]. Continuous variables are expressed as mean ± standard deviation (SD) when normally distributed or median (interquartile range [IQR]) when non-normally distributed and are compared using t-test or Mann Whitney U test as appropriate. Categorical variables are expressed as numbers and percentages and were compared using the Fisher exact test or Chi-square test as appropriate. Multiple linear regression analysis was conducted to examine the variables independently associated with PMI. Survival from all-cause death and ADHF re-hospitalization for 1000 days after discharge was analyzed. Kaplan-Meier curves of survival and ADHF re-hospitalization were drawn and analyzed by log-rank test for the study population divided by the lowest gender-based quartile of the PMI. The prognostic impact of low PMI on all-cause death and ADHF re-hospitalization were assessed using multivariate Cox proportional hazards regression analysis. Multivariate analysis using the following variables; age, gender, body mass index (BMI), history of heart failure admission, ischemic etiology, atrial fibrillation, systolic blood pressure, N-terminal pro B-type natriuretic peptide (NT-proBNP), hemoglobin, estimated glomerular filtration rate (eGFR) and LVEF, calculated the Hazard ratios (HR) and 95% confidence intervals (CI). Values of P <0.05 were considered to indicate statistical significance. Statistical analyses were performed using JMP version 12.2 (SAS Institute, Cary, NC).

## Results

**Fig 1:**
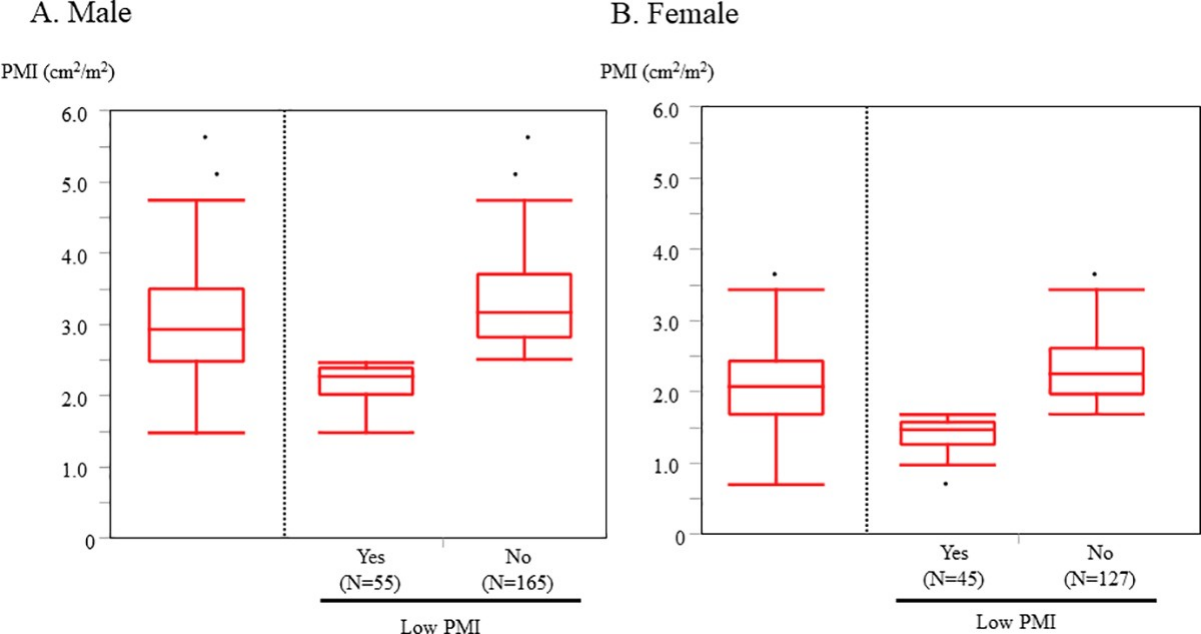
The distribution of PMI. Low PMI was defined as the lowest gender-based quartile of the PMI. Cutoff value 2.47 (cm^2^/m^2^) for male and 1.68 (cm^2^/m^2^) for female) **A.** Male **B.** Female. PMI, psoas muscle index.

Study flowchart is shown in S2 Fig. Among 865 elderly patients with ADHF, 470 patients who did not undergo abdominal CT and 3 patients without data of height were excluded. Finally, 392 subjects were available for analysis. The reasons for the performance of CT scan are shown in S3 Fig. The median age was 79 years (IQR 74–85) and 56% were male. The median follow-up period was 843 days (IQR 339–1360). The median and mean PMI were 2.94 and 3.03 (cm^2^/m^2^) in male and 2.08 and 2.08 (cm^2^/m^2^) in female, respectively (Fig 1).

Table 1 shows the baseline characteristics at admission of the overall study population and the participants divided into low PMI and high PMI groups according to the lowest gender-based quartile of the PMI (cutoff value 2.47 [cm^2^/m^2^] for male and 1.68 [cm^2^/m^2^] for female). In male low PMI group was associated with lower BMI, lower systolic blood pressure, lower hemoglobin level, higher NT-proBNP level and lower LVEF compared with high PMI group. On the other hand, in female low PMI group was associated with lower BMI, lower diastolic blood pressure and higher E/e’ compared with high PMI group. There were no significant differences in the percentage of patients with the comorbidities such as hypertension, diabetes mellitus, or dyslipidemia etc. between the two groups. Although there was no significant difference in in-hospital mortality between the 2 groups, length of hospital stay was significantly longer in low PMI group compared with high PMI group.

**Table 1:** Baseline characteristics of study population at admission.

			Male low PMI				Female low PMI		
		Male Overall	No (n = 165)	Yes (n = 55)	P value	Female Overall	No (n = 127)	Yes (n = 45)	P value
	Age, years	79 (73–83)	78 (73–83)	79 (73–86)	0.14	82 (75–86)	82 (75–87)	80 (74–85)	0.25
	BMI, (kg/m^2^)	23.5 (21.4–26.1)	24.2 (22.1–26.5)	21.7 (18.9–24.5)	<0.001	21.5 (18.9–24.3)	21.8 (19.9–24.6)	19.2 (18.0–22.6)	0.001
	BSA (m^2^)	1.69±0.15	1.71±0.14	1.63±0.17	0.002	1.42±0.15	1.44±0.15	1.39±0.15	0.11
	NYHA, (I/II/III/IV) (%)	0/62/75/83 (0/28/34/38)	0/51/56/58 (0/31/34/35)	0/11/19/25 (0/20/35/45)	0.23	0/45/56/71 (0/26/33/41)	0/35/38/54 (0/28/30/42)	0/10/18/17 (0/22/40/38)	0.45
Etiology					0.063				0.38
	IHD	87 (40)	68 (41)	19 (35)		29 (17)	25 (20)	4 (8.9)	
	DCM	21 (10)	12 (7.3)	9 (16)		9 (5.2)	7 (5.5)	2 (4.4)	
	VHD	80 (36)	57 (35)	23 (42)		94 (55)	66 (52)	28 (62)	
Past history and comorbidities									
	Smoking history, n (%)	161 (74)	126 (77)	35 (64)	0.055	31 (18)	23 (18)	8 (18)	0.96
	Previous HF hospitalization, n (%)	80 (36)	58 (35)	22 (40)	0.52	63 (37)	50 (40)	13 (29)	0.20
	Atrial fibrillation, n (%)	120 (55)	92 (56)	28 (51)	0.53	95 (55)	73 (57)	22 (49)	0.32
	Hypertension, n (%)	141 (64)	107 (65)	34 (62)	0.69	113 (66)	86 (68)	27 (60)	0.35
	Diabetes mellitus, n (%)	79 (36)	59 (36)	20 (36)	0.94	50 (29)	37 (29)	13 (29)	0.98
	Dyslipidemia, n (%)	96 (44)	72 (44)	24 (44)	1.00	74 (43)	54 (43)	20 (44)	0.82
	Previous stroke, TIA, n (%)	45 (21)	38 (23)	7 (13)	0.11	17 (10)	12 (10)	5 (11)	0.78
	COPD, n (%)	11 (5.0)	10 (6.1)	1 (1.8)	0.30	1 (0.6)	1 (0.8)	0 (0)	1.00
	Hemodialysis, n (%)	4 (1.8)	3 (1.8)	1 (1.8)	1.00	(0)	0 (0)	0 (0)	
	Internal pacemaker, n (%)	14 (6.4)	11 (6.7)	3 (5.5)	1.00	22 (13)	17 (13)	5 (11)	0.69
	ICD, n (%)	17 (7.7)	11 (6.7)	6 (11)	0.38	4 (2.3)	3 (2.4)	1 (2.2)	1.00
Vital signs									
	SBP, mmHg	138 (116–154)	140 (120–157)	128 (108–147)	0.009	135 (120–152)	135 (120–153)	134 (120–151)	0.92
	DBP, mmHg	76 (63–90)	76 (64–92)	76 (60–88)	0.53	74 (65–90)	76 (67–92)	68 (60–89)	0.030
	Heart rate, (bpm)	81 (70–102)	80 (70–103)	82 (70–102)	0.75	90 (77–117)	89 (78–117)	90 (74–117)	0.76
Laboratory data									
	Hemoglobin, (g/dl)	11.6±2.1	11.8±2.0	11.1±2.3	0.040	11.1±1.9	11.1±2.0	11.3±1.8	0.58
	Serum albumin, (mg/dl)	3.7 (3.4–4.0)	3.8 (3.4–4.0)	3.7 (3.2–3.9)	0.18	3.6 (3.1–3.9)	3.5 (3.2–3.8)	3.6 (3.1–3.9)	0.84
	eGFR, (ml/min/1.73 m^2^)	47 (34–63)	48 (34–64)	44 (32–60)	0.34	49 (34–63)	47 (34–62)	53 (37–67)	0.28
	NT-proBNP, (pg/ml)	4123 (2112–9696)	3761 (1948–7645)	6667 (2747–15657)	0.008	4199 (1961–7083)	4140 (1982–6679)	4440 (1736–9801)	0.35
Echocardiography									
	LVEF, (%)	45 (32–57)	47 (36–57)	36 (28–56)	0.012	55 (37–62)	53 (37–61)	56 (37–64)	0.44
	rEF / mrEF / pEF	88 / 37 / 95 (40% /17% / 43%)	57 / 30 / 78 (35% / 18% / 47%)	31 / 7 / 17 (56% / 13% / 31%)	0.018	51 / 14 / 106 (30% / 8% / 62%)	39 / 10 / 78 (31% / 8% / 61%)	12 / 4 / 28 (27% / 9% / 64%)	0.90
	LVEDD, (mm)	55 (49–60)	55 (49–60)	55 (47–60)	0.88	45 (40–51)	45 (40–51)	47 (40–52)	0.39
	LVESD, (mm)	41 (33–50)	40 (33–48)	43 (33–52)	0.20	32 (27–39)	31 (27–39)	33 (28–40)	0.29
	LAD, (mm)	46 (40–53)	46 (41–53)	44 (39–50)	0.24	45 (40–50)	45 (40–50)	44 (40–52)	0.82
	E/e’	19.2 (13.9–28.8)	20.5 (14.0–28.8)	18.0 (13.8–27.4)	0.30	24.4 (17.0–36.3)	22.9 (16.8–30.9)	34.8 (22.7–41.8)	0.001
In-hospital outcome									
	Length of hospital stay (days)	16 (11–25)	16 (11–24)	19 (13–29)	0.064	17 (11–23)	16 (10–22)	18 (14–25)	0.023
	In-hospital death, n (%)	8 (3.6)	4 (2.4)	4 (7.3)	0.11	5 (2.9)	4 (3.2)	1 (2.2)	1.00
Medication at discharge									
	Loop diuretics, n (%)	168 (79)	125 (78)	43 (84)	0.31	129 (77)	95 (77)	34 (77)	0.99
	Thiazide, n (%)	25 (14)	20 (15)	5 (13)	0.74	12 (8.8)	10 (9.5)	2 (6.5)	0.73
	ACEI/ARB, n (%)	127 (60)	92 (57)	35 (69)	0.14	89 (53)	66 (54)	23 (52)	0.87
	β-blocker, n (%)	158 (75)	117 (73)	41 (80)	0.27	131 (78)	93 (76)	38 (86)	0.14
	MRA, n (%)	74 (35)	57 (35)	17 (33)	0.79	48 (29)	33 (27)	15 (34)	0.36
	CCB, n (%)	64 (30)	52 (32)	12 (24)	0.23	68 (41)	51 (41)	17 (39)	0.74
	Statins, n (%)	83 (39)	63 (39)	20 (39)	0.99	54 (32)	41 (33)	13 (30)	0.64
	Aspirin, n (%)	115 (54)	90 (56)	25 (49)	0.39	70 (42)	55 (45)	15 (34)	0.22

PMI, psoas muscle index; BMI, body mass index; BSA, body surface area; NYHA, New York Heart Association, IHD, ischemic heart disease; DCM, dilated cardiomyopathy; VHD; valvular heart disease; HF, heart failure; TIA, transient ischemic attack; COPD, chronic obstructive pulmonary disease; ICD, implantable cardioverter defibrillator; SBP, systolic blood pressure; DBP, diastolic blood pressure; eGFR, estimated glomerular filtration rate; NT-proBNP, N-terminal pro B-type natriuretic peptide; LVEF, left ventricular ejection fraction; rEF. reduced ejection fraction; mrEF, midrange ejection fraction; pEF, preserved ejection fraction; LVEDD, left ventricular end-diastolic dimension; LVESD, left ventricular end-systolic dimension; LAD, left atrial dimension; ACEI, angiotensin-converting enzyme inhibitor; ARB, angiotensin receptor blocker; MRA, mineralocorticoid receptor antagonist; CCB, calcium channel blocker.

### The variables associated with PMI

**Fig 2:**
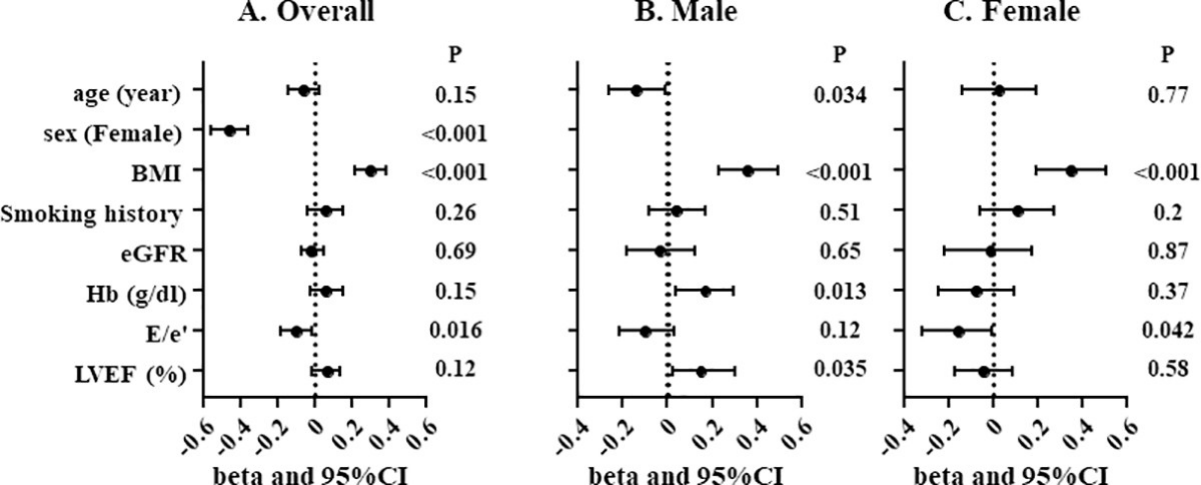
Multiple linear regression analysis for PMI. Overall population (A), male (B) and female (C). PMI, psoas muscle index; BMI, body mass index; eGFR, estimated glomerular filtration rate; Hb, hemoglobin level; LVEF, left ventricular ejection fraction.

Multiple linear regression analysis revealed female sex, BMI and E/e’, but not LVEF, were independently associated with PMI in overall population. Whereas age, BMI, hemoglobin level and LVEF were independently associated with PMI in male, BMI and E/e’ were independently associated with PMI in female (Fig 2).

### PMI and future adverse events

**Fig 3:**
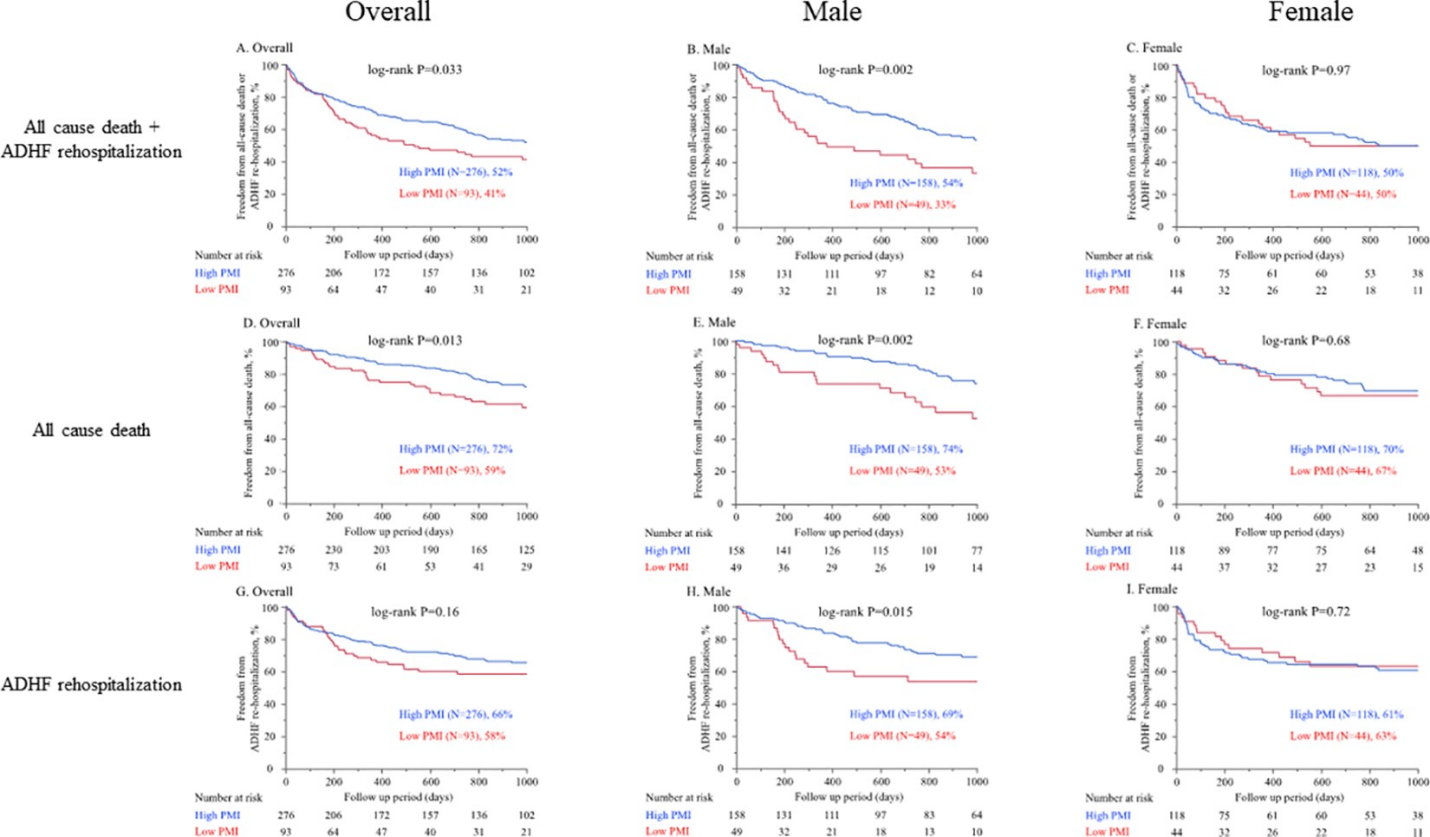
Kaplan-Meier survival estimates according to the lowest gender-based quartile of the PMI. Cutoff value 2.47 (cm^2^/m^2^) for male and 1.68 (cm^2^/m^2^) for female. In **A** to **C** the endpoint was defined as the composite of all-cause death and ADHF re-hospitalization. (**A.** Overall, **B.** Male, **C.** Female). In **D** to **F** the endpoint was defined as all-cause death. (**D.** Overall, **E.** Male, **F.** Female). In **G** to **I** the endpoint was defined as ADHF re-hospitalization. (**G.** Overall, **H.** Male, **I.** Female). PMI, psoas muscle index; ADHF, acute decompensated heart failure.

During 843 (339–1000) days follow-up period after discharge, 98 (27%) all-cause death including 52 (14%) cardiac death and 117 (32%) ADHF rehospitalization occurred. Kaplan-Meier survival estimates according to the lowest gender-based quartile of the PMI are shown in Fig 3. Low PMI was associated with a higher rate of the primary endpoint defined as the composite of all-cause death and ADHF re-hospitalization in overall population (P = 0.033, log-rank test, Fig 3A), and male (P = 0.002, Fig 3B), but not in female (P = 0.97, Fig 3C). The same trend was observed regarding all-cause death (overall, P = 0.013; male, P = 0.002; female, P = 0.68, Fig 3D–3F) and cardiac death (overall, P = 0.005; male, P = 0.007; female P = 0.23). On the other hand, low PMI was associated with higher rate of ADHF re-hospitalization only in male (P = 0.015, log-rank test, Fig 3H), but not in overall (P = 0.16, log-rank test, Fig 3G) or female (P = 0.72, log-rank test, Fig 3I).

Multivariate Cox proportional hazards analysis was conducted to identify independent predictors for the primary endpoint defined as the composite of all-cause death and ADHF re-hospitalization. We found low PMI was an independent predictor of primary endpoint in overall (HR: 1.52, 95% CI: 1.06–2.16, P = 0.024) and male (HR: 1.85, 95% CI: 1.13–2.95, P = 0.015), but not in female. (HR: 1.25, 95% CI: 0.68–2.25, P = 0.46, Table 2). On the other hand, BMI, which is an index of body mass but not body composition, failed to predict the primary endpoint (Table 2).

**Table 2:** Univariate-unadjusted and multivariate-adjusted Cox proportional hazard model analysis for the composite endpoint of all-cause death and ADHF re-hospitalization.

**A. Overall**						
	Univariate-unadjusted			Multivariate-adjusted		
	HR	95%CI	P-value	HR	95%CI	P-value
Age	1.04	1.02–1.06	<0.001	1.05	1.02–1.07	<0.001
Sex (Female)	1.15	0.85–1.55	0.36	1.12	0.79–1.57	0.52
BMI	0.97	0.93–1.01	0.15	1.01	0.96–1.06	0.65
History of heart failure admission	2.65	1.96–3.58	<0.001	2.29	1.63–3.22	<0.001
Ischemic etiology	1.24	0.90–1.69	0.19	1.24	0.85–1.81	0.26
Atrial fibrillation	1.59	1.17–2.18	0.003	1.48	1.06–2.08	0.022
Systolic blood pressure	0.99	0.98–0.99	0.003	0.99	0.99–1.00	0.21
NT-proBNP	1.00	0.99–1.00	0.19	1.00	0.99–1.00	0.40
hemoglobin	0.89	0.82–0.95	0.001	0.96	0.88–1.05	0.37
eGFR	0.99	0.98–0.99	0.014	0.99	0.99–1.01	0.96
LVEF	0.99	0.98–1.00	0.10	0.99	0.98–1.00	0.16
Low PMI	1.42	1.02–1.96	0.038	1.52	1.06–2.16	0.024
**B. Male**						
	Univariate-unadjusted			Multivariate-adjusted		
	HR	95%CI	P-value	HR	95%CI	P-value
Age	1.04	1.01–1.07	0.009	1.05	1.02–1.09	0.002
BMI	0.97	0.91–1.03	0.29	1.01	0.94–1.07	0.84
History of ADHF admission	2.65	1.78–3.97	<0.001	2.59	1.60–4.21	<0.001
Ischemic etiology	1.38	0.92–2.06	0.12	1.26	0.79–2.01	0.33
Atrial fibrillation	1.74	1.15–2.65	0.008	1.51	0.97–2.41	0.071
Systolic blood pressure	0.99	0.98–0.99	0.009	0.99	0.99–1.00	0.21
NT-proBNP	1.00	0.99–1.00	0.37	1.00	0.99–1.00	0.47
hemoglobin	0.89	0.81–0.97	0.012	0.47	0.11–2.07	0.32
eGFR	0.99	0.98–1.01	0.33	1.01	0.99–1.02	0.18
LVEF	0.99	0.98–1.00	0.15	0.99	0.98–1.01	0.47
Low PMI	1.98	1.27–3.03	0.003	1.85	1.13–2.95	0.015
**C. Female**						
	Univariate-unadjusted			Multivariate-adjusted		
	HR	95%CI	P-value	HR	95%CI	P-value
Age	1.05	1.02–1.08	0.004	1.04	1.00–1.08	0.042
BMI	0.98	0.92–1.04	0.53	1.01	0.94–1.09	0.78
History of ADHF admission	2.63	1.67–4.16	<0.001	2.16	1.26–3.72	0.005
Ischemic etiology	1.21	0.66–2.07	0.51	1.17	0.58–2.27	0.64
Atrial fibrillation	1.45	0.92–2.34	0.11	1.41	0.84–2.43	0.20
Systolic blood pressure	0.99	0.98–1.00	0.11	0.99	0.99–1.01	0.56
NT-proBNP	1.00	0.99–1.00	0.12	1.00	0.99–1.00	0.94
hemoglobin	0.88	0.79–0.99	0.040	0.97	0.84–1.12	0.68
eGFR	0.98	0.97–0.99	0.007	0.99	0.98–1.00	0.20
LVEF	0.99	0.98–1.01	0.24	0.99	0.97–1.01	0.24
Low PMI	0.99	0.59–1.60	0.97	1.25	0.68–2.25	0.46

ADHF, acute decompensated heart failure; HR, hazard ratio; CI, confidence interval; BMI, body mass index; NT-proBNP, N-terminal pro-brain natriuretic peptide; eGFR, estimated glomerular filtration rate; LVEF, left ventricular ejection fraction; PMI, psoas muscle index.

## Discussion

The main findings of the present study were as follows: 1) PMI was independently associated with female sex, BMI and E/e’. 2) Low PMI defined as the lowest gender-based quartile of the PMI was associated with a higher rate of the primary endpoint defined as the composite of all-cause death and ADHF re-hospitalization in male. 3) By Cox proportional hazard model analysis, low PMI, but not BMI was an independent predictor of the primary endpoint. From these findings we concluded that low PMI was associated with clinical adverse events after discharge in male elderly patients with HF.

### PMI as a novel index of muscle mass

There have been a lot of measures developed for evaluating skeletal muscle mass such as the bioelectrical impedance analysis (BIA) and dual energy X-ray absorptiometry (DEXA) [5, 21, 22]. However, because of various approaches or cut-off values employed for its definition, they have not yet been standardized [5, 21–31]. To seek the simple and reliable measures of skeletal muscle mass, our approach, the PMA measurement by CT can be quantitative and easy to evaluate by non-contrast CT. Of note, the PMA obtained from CT has been shown to well correlate with whole body muscle mass [15]. In recent studies, clinical prognostic value of PMA has been validated in the different cohorts [16, 17]. Thus, we hypothesized that PMA could be applied as a surrogate of total muscle mass in HF population and we sought to explore its prognostic significance.

### Association of lower PMI with HF

In the present study, female sex and lower BMI were independently associated with lower PMI (Fig 2), which was consistent with the previous report [15]. Also, we found E/e’ were negatively associated with PMI (Fig 2). This finding is in line with the previous reports that showed negative correlation of E/e’ with skeletal muscle mass index (SMI) assessed by BIA or appendicular skeletal muscle mass by DEXA [32, 33]. As a potential mechanism, elevated LV end-diastolic pressure can cause pulmonary congestion accompanied by oxygen desaturation on exertion or even at rest, which can eventually lead to the decreased physical activity. A sequence of these physiological responses may result in disuse muscle atrophy in elderly patients with HF. On the other hand, lower muscle mass is related to insulin resistance [34] which can cause the exacerbation of diastolic dysfunction [32]. Collectively, LV diastolic dysfunction can potentially lead to decreased muscle mass and vice versa. Their causal relationship and detailed mechanisms remain unproved and need further investigation.

### The potential of muscle mass measurement in HF

Our findings of higher cumulative incidence of all cause death and ADHF re-hospitalization in low PMI group were consistent with the previous reports [14]. In the present study PMI was not associated with future clinical adverse events in female. The differential clinical impact of PMI between male and female remains unknown. The gender difference in the clinical impact of PMI in the other cohort has been inconsistent [35, 36]. In the present study female showed much lower PMI values and their distribution was narrower compared with male. Also, in female age was higher than male and the elderly-related factors such as comorbidities, cognitive impairment, poor adherence, or social circumstances might additionally influence the clinical outcome.

One recent single center observational study enrolling patients with HFrEF reported low PMA was associated with a higher rate of all-cause mortality at 1 year only in males and those under 75 years old [35]. However, in their study, the risk of bias due to relatively small sample size (N = 160) and no correction of PMA by body surface area or height cannot be excluded. The present study with larger sample size comprehensively enrolled all types of HF (i.e. HFpEF) and employed PMA corrected by the square of height (PMI). Thus, we believe that the present study assessed the prognostic value of PMA in elderly HF patients in a more appropriate manner.

Interestingly, BMI, a measure of body mass but not composition, did not remain significant for the prediction of the clinical outcome in the present study (Table 2), although low BMI has been shown to be associated with unfavorable outcome in patients with CHF, often referred to as “obesity paradox” [37, 38]. Our findings raise the hypothesis that increased muscle mass in obese patients might explain one of the potential mechanisms of obesity paradox. This hypothesis is also supported by the previous study, which demonstrated that midarm muscle area but not BMI predicted long-term survival in elderly patients with CHF [39]. Taken together, PMA measurement might have a potential for providing better prediction of future adverse events compared with BMI. These findings might be linked to the identification of the patients who are at high risk for repeated hospitalization, often referred to as “frequent flyer” [40]. It was shown that exercise training increased whole-body lean tissue mass in elderly patients and was associated with a reduced hospitalization rate and an improved health-related quality of life in patients with CHF [41, 42]. We believe that from our findings PMI may help identify patients who will greatly benefit from exercise training. However, it is also possible that low PMI and/or its clinical impact might be irreversible any longer and this patient group might need other interventions like a nutrition therapy or further optimization of medical therapy. Further investigation will be needed to elucidate this issue.

### Limitations

This study has several important limitations. First, the standard procedure for the assessment muscle mass such as BIA or DEXA was not assessed in this study. Since PMA obtained from CT has been shown to correlate well with whole body muscle mass [15], we utilized PMA as a surrogate for total muscle mass, but in a small study their correlation was modest [43]. Second, this study only included the patients who underwent abdominal CT. Although we verified the data of baseline characteristics and the clinical outcome after discharge between CT group and non-CT group (S1 Table and S4 Fig). Also, patients who underwent CT scans during hospitalization and/or up to 1 year before hospitalization were included. There may have been some changes in muscle mass during this time interval, although we verified the data in some patients who underwent multiple CT scans within 1 year before hospitalization and they scarcely showed the changes in PMI in the time interval (data not shown). Third, although low PMI assessed by our method corresponds with pre-sarcopenia that was defined as a decrease in muscle mass [2], we did not evaluate the other objective parameters of sarcopenia indicating muscle or physical function such as handgrip strength gait speed, or exercise tests of METS questionnaire. Fourth, although sarcopenia is linked to the pathology of frailty [13] and cachexia [44], they were not assessed in the present study. Lastly, the present study was conducted at a single center with a relatively small number of patients with homogeneous Asian population, the generalizability of these results to other ethnic groups may be limited. To confirm the results of the present study, a prospective multicenter study is needed.

## Conclusions

In conclusion, PMI, a novel marker of muscle mass, predicted clinical adverse events in male elderly patients hospitalized for ADHF. Further studies are needed to assess whether low skeletal muscle mass can be a potential therapeutic target to improve the outcome of ADHF patients especially for frequent flyers.

## Supporting information

S1 Fig An example of tracing of psoas muscles on CT scan. **A.** axial section **B.** sagittal section of CT scan. As shown in the yellow line, the bilateral psoas muscles at the level of the fourth lumbar vertebra were manually traced. CT, computed tomography.
(TIF)

S2 Fig Study flowchart of the patient enrollment and follow up. The patients who were hospitalized at the Sakakibara Heart Institute between November 2011 and December 2015 were enrolled. Those who did not undergo abdominal CT during or within 1 year before hospitalization and no height data were excluded. ADHF, acute decompensated heart failure; CT, computed tomography.
(TIF)

S3 Fig The reasons why CT was performed in the study population. The regions or diseases of interest are shown. The most common reason was evaluation of lung field. CT, computed tomography.
(TIF)

S4 Fig Kaplan-Meier curve according to the CT group and non-CT group. The endpoint was defined as the composite of all-cause death and ADHF re-hospitalization (**A.**), all-cause death (**B.**) and ADHF re-hospitalization (**C.**). ADHF, acute decompensated heart failure; CT, computed tomography.
(TIF)

S1 Table Baseline characteristics of CT group and non-CT group. (PDF)

## References

[1] Ambrosy AP, Fonarow GC, Butler J, Chioncel O, Greene SJ, et al (2014) The global health and economic burden of hospitalizations for heart failure: lessons learned from hospitalized heart failure registries. J Am Coll Cardiol 63: 1123–1133. 10.1016/j.jacc.2013.11.053 24491689

[2] Cruz-Jentoft AJ, Baeyens JP, Bauer JM, Boirie Y, Cederholm T, et al (2010) Sarcopenia: European consensus on definition and diagnosis: Report of the European Working Group on Sarcopenia in Older People. Age Ageing 39: 412–423. 10.1093/ageing/afq034 20392703 PMC2886201

[3] Muscaritoli M, Anker SD, Argilés J, Aversa Z, Bauer JM, et al (2010) Consensus definition of sarcopenia, cachexia and pre-cachexia: Joint document elaborated by Special Interest Groups (SIG) “cachexia-anorexia in chronic wasting diseases” and “nutrition in geriatrics”. Clinical Nutrition 29: 154–159. 10.1016/j.clnu.2009.12.004 20060626

[4] Fielding RA, Vellas B, Evans WJ, Bhasin S, Morley JE, et al (2011) Sarcopenia: an undiagnosed condition in older adults. Current consensus definition: prevalence, etiology, and consequences. International working group on sarcopenia. J Am Med Dir Assoc 12: 249–256. 10.1016/j.jamda.2011.01.003 21527165 PMC3377163

[5] Janssen I, Heymsfield SB, Ross R (2002) Low relative skeletal muscle mass (sarcopenia) in older persons is associated with functional impairment and physical disability. J Am Geriatr Soc 50: 889–896. 10.1046/j.1532-5415.2002.50216.x 12028177

[6] Kim NH, Kim HS, Eun CR, Seo JA, Cho HJ, et al (2011) Depression is associated with sarcopenia, not central obesity, in elderly korean men. J Am Geriatr Soc 59: 2062–2068. 10.1111/j.1532-5415.2011.03664.x 22092258

[7] Malmstrom TK, Miller DK, Herning MM, Morley JE (2013) Low appendicular skeletal muscle mass (ASM) with limited mobility and poor health outcomes in middle-aged African Americans. J Cachexia Sarcopenia Muscle 4: 179–186. 10.1007/s13539-013-0106-x 23532635 PMC3774914

[8] Szulc P, Beck TJ, Marchand F, Delmas PD (2005) Low skeletal muscle mass is associated with poor structural parameters of bone and impaired balance in elderly men—the MINOS study. J Bone Miner Res 20: 721–729. 10.1359/JBMR.041230 15824844

[9] Carter ND, Khan KM, Mallinson A, Janssen PA, Heinonen A, et al (2002) Knee extension strength is a significant determinant of static and dynamic balance as well as quality of life in older community-dwelling women with osteoporosis. Gerontology 48: 360–368. 10.1159/000065504 12393951

[10] Landi F, Cruz-Jentoft AJ, Liperoti R, Russo A, Giovannini S, et al (2013) Sarcopenia and mortality risk in frail older persons aged 80 years and older: results from ilSIRENTE study. Age Ageing 42: 203–209. 10.1093/ageing/afs194 23321202

[11] Mancini DM, Walter G, Reichek N, Lenkinski R, McCully KK, et al (1992) Contribution of skeletal muscle atrophy to exercise intolerance and altered muscle metabolism in heart failure. Circulation 85: 1364–1373. 10.1161/01.cir.85.4.1364 1555280

[12] Fulster S, Tacke M, Sandek A, Ebner N, Tschope C, et al (2013) Muscle wasting in patients with chronic heart failure: results from the studies investigating co-morbidities aggravating heart failure (SICA-HF). Eur Heart J 34: 512–519. 10.1093/eurheartj/ehs381 23178647

[13] Afilalo J, Alexander KP, Mack MJ, Maurer MS, Green P, et al (2014) Frailty assessment in the cardiovascular care of older adults. J Am Coll Cardiol 63: 747–762. 10.1016/j.jacc.2013.09.070 24291279 PMC4571179

[14] Narumi T, Watanabe T, Kadowaki S, Takahashi T, Yokoyama M, et al (2015) Sarcopenia evaluated by fat-free mass index is an important prognostic factor in patients with chronic heart failure. Eur J Intern Med 26: 118–122. 10.1016/j.ejim.2015.01.008 25657117

[15] Shen W, Punyanitya M, Wang Z, Gallagher D, St-Onge MP, et al (2004) Total body skeletal muscle and adipose tissue volumes: estimation from a single abdominal cross-sectional image. J Appl Physiol 97: 2333–2338. 10.1152/japplphysiol.00744.2004 15310748

[16] Saji M, Lim DS, Ragosta M, LaPar DJ, Downs E, et al (2016) Usefulness of Psoas Muscle Area to Predict Mortality in Patients Undergoing Transcatheter Aortic Valve Replacement. Am J Cardiol 118: 251–257. 10.1016/j.amjcard.2016.04.043 27236254

[17] Zuckerman J, Ades M, Mullie L, Trnkus A, Morin JF, et al (2017) Psoas Muscle Area and Length of Stay in Older Adults Undergoing Cardiac Operations. Ann Thorac Surg 103: 1498–1504. 10.1016/j.athoracsur.2016.09.005 27863730

[18] Yancy CW, Jessup M, Bozkurt B, Butler J, Casey DE, et al (2013) 2013 ACCF/AHA Guideline for the Management of Heart Failure. Circulation 128: e240–e327. 10.1161/CIR.0b013e31829e8776 23741058

[19] Okamura H, Kimura N, Tanno K, Mieno M, Matsumoto H, et al (2018) The impact of preoperative sarcopenia, defined based on psoas muscle area, on long-term outcomes of heart valve surgery. J Thorac Cardiovasc Surg. 10.1016/j.jtcvs.2018.06.098 30139644

[20] Ponikowski P, Voors AA, Anker SD, Bueno H, Cleland JGF, et al (2016) 2016 ESC Guidelines for the diagnosis and treatment of acute and chronic heart failure: The Task Force for the diagnosis and treatment of acute and chronic heart failure of the European Society of Cardiology (ESC)Developed with the special contribution of the Heart Failure Association (HFA) of the ESC. Eur Heart J 37: 2129–2200. 10.1093/eurheartj/ehw128 27206819

[21] Chien MY, Huang TY, Wu YT (2008) Prevalence of sarcopenia estimated using a bioelectrical impedance analysis prediction equation in community-dwelling elderly people in Taiwan. J Am Geriatr Soc 56: 1710–1715. 10.1111/j.1532-5415.2008.01854.x 18691288

[22] Janssen I, Baumgartner RN, Ross R, Rosenberg IH, Roubenoff R (2004) Skeletal muscle cutpoints associated with elevated physical disability risk in older men and women. Am J Epidemiol 159: 413–421. 10.1093/aje/kwh058 14769646

[23] Delmonico MJ, Harris TB, Lee JS, Visser M, Nevitt M, et al (2007) Alternative definitions of sarcopenia, lower extremity performance, and functional impairment with aging in older men and women. J Am Geriatr Soc 55: 769–774. 10.1111/j.1532-5415.2007.01140.x 17493199

[24] Newman AB, Kupelian V, Visser M, Simonsick E, Goodpaster B, et al (2003) Sarcopenia: alternative definitions and associations with lower extremity function. J Am Geriatr Soc 51: 1602–1609. 10.1046/j.1532-5415.2003.51534.x 14687390

[25] Schaap LA, Pluijm SM, Deeg DJ, Visser M (2006) Inflammatory markers and loss of muscle mass (sarcopenia) and strength. Am J Med 119: 526 e529-517. 10.1016/j.amjmed.2005.10.049 16750969

[26] Melton LJ III, Khosla S, Crowson CS, O’connor MK, O’fallon WM, et al (2000) Epidemiology of sarcopenia. J Am Geriatr Soc 48: 625–630. 10855597

[27] Tank LB, Movsesyan L, Mouritzen U, Christiansen C, Svendsen OL (2002) Appendicular lean tissue mass and the prevalence of sarcopenia among healthy women. Metabolism 51: 69–74. 10.1053/meta.2002.28960 11782875

[28] Baumgartner RN, Koehler KM, Gallagher D, Romero L, Heymsfield SB, et al (1998) Epidemiology of sarcopenia among the elderly in New Mexico. Am J Epidemiol 147: 755–763. 10.1093/oxfordjournals.aje.a009520 9554417

[29] Castillo EM, Goodman-Gruen D, Kritz-Silverstein D, Morton DJ, Wingard DL, et al (2003) Sarcopenia in elderly men and women: the Rancho Bernardo study. Am J Prev Med 25: 226–231. 10.1016/s0749-3797(03)00197-1 14507529

[30] Gillette-Guyonnet S, Nourhashemi F, Andrieu S, Cantet C, Albarede JL, et al (2003) Body composition in French women 75+ years of age: the EPIDOS study. Mech Ageing Dev 124: 311–316. 10.1016/s0047-6374(02)00198-7 12663128

[31] Iannuzzi-Sucich M, Prestwood KM, Kenny AM (2002) Prevalence of sarcopenia and predictors of skeletal muscle mass in healthy, older men and women. J Gerontol A Biol Sci Med Sci 57: M772–777. 10.1093/gerona/57.12.m772 12456735

[32] Ko B-J, Chang Y, Kang JG, Kim J, Jung H-S, et al (2018) Low relative muscle mass and left ventricular diastolic dysfunction in middle-aged adults. Int J Cardiol 255: 118–123. 10.1016/j.ijcard.2017.07.089 29425549

[33] Bekfani T, Pellicori P, Morris DA, Ebner N, Valentova M, et al (2016) Sarcopenia in patients with heart failure with preserved ejection fraction: Impact on muscle strength, exercise capacity and quality of life. Int J Cardiol 222: 41–46. 10.1016/j.ijcard.2016.07.135 27454614

[34] Kalyani RR, Corriere M, Ferrucci L (2014) Age-related and disease-related muscle loss: the effect of diabetes, obesity, and other diseases. The Lancet Diabetes & Endocrinology 2: 819–829. 24731660 10.1016/S2213-8587(14)70034-8 PMC4156923

[35] Lopez PD, Nepal P, Akinlonu A, Nekkalapudi D, Kim K, et al (2019) Low Skeletal Muscle Mass Independently Predicts Mortality in Patients with Chronic Heart Failure after an Acute Hospitalization. Cardiology 142: 28–36. 10.1159/000496460 30893691

[36] Mamane S, Mullie L, Piazza N, Martucci G, Morais J, et al (2016) Psoas Muscle Area and All-Cause Mortality After Transcatheter Aortic Valve Replacement: The Montreal-Munich Study. Can J Cardiol 32: 177–182. 10.1016/j.cjca.2015.12.002 26821840

[37] Horwich TB, Fonarow GC, Hamilton MA, MacLellan WR, Woo MA, et al (2001) The relationship between obesity and mortality in patients with heart failure. J Am Coll Cardiol 38: 789–795. 10.1016/s0735-1097(01)01448-6 11527635

[38] Fonarow GC, Srikanthan P, Costanzo MR, Cintron GB, Lopatin M, et al (2007) An obesity paradox in acute heart failure: analysis of body mass index and inhospital mortality for 108,927 patients in the Acute Decompensated Heart Failure National Registry. Am Heart J 153: 74–81. 10.1016/j.ahj.2006.09.007 17174642

[39] Casas-Vara A, Santolaria F, Fernández-Bereciartúa A, González-Reimers E, García-Ochoa A, et al (2012) The obesity paradox in elderly patients with heart failure: Analysis of nutritional status. Nutrition 28: 616–622. 10.1016/j.nut.2011.10.006 22261572

[40] Bakal JA, McAlister FA, Liu W, Ezekowitz JA (2014) Heart failure re-admission: measuring the ever shortening gap between repeat heart failure hospitalizations. PLoS One 9: e106494 10.1371/journal.pone.0106494 25211034 PMC4161342

[41] Candow DG, Chilibeck PD, Facci M, Abeysekara S, Zello GA (2006) Protein supplementation before and after resistance training in older men. Eur J Appl Physiol 97: 548–556. 10.1007/s00421-006-0223-8 16767436

[42] Belardinelli R, Georgiou D, Cianci G, Purcaro A (1999) Randomized, controlled trial of long-term moderate exercise training in chronic heart failure: effects on functional capacity, quality of life, and clinical outcome. Circulation 99: 1173–1182. 10.1161/01.cir.99.9.1173 10069785

[43] Fujimoto K, Inage K, Eguchi Y, Orita S, Toyoguchi T, et al (2019) Dual-Energy X-ray Absorptiometry and Bioelectrical Impedance Analysis are Beneficial Tools for Measuring the Trunk Muscle Mass of Patients with Low Back Pain. Spine Surg Relat Res 3: 335–341. 10.22603/ssrr.2018-0040 31768453 PMC6834466

[44] Evans WJ, Morley JE, Argilés J, Bales C, Baracos V, et al (2008) Cachexia: a new definition. Clin Nutr 27: 793–799. 10.1016/j.clnu.2008.06.013 18718696

