# Supplementary figures and images for: Low muscle mass assessed by psoas muscle area is associated with clinical adverse events in elderly patients with heart failure
Source: PLoS One. 2021 Feb 16;16(2):e0247140. doi: 10.1371/journal.pone.0247140 (PMC7886171; doi:10.1371/journal.pone.0247140)

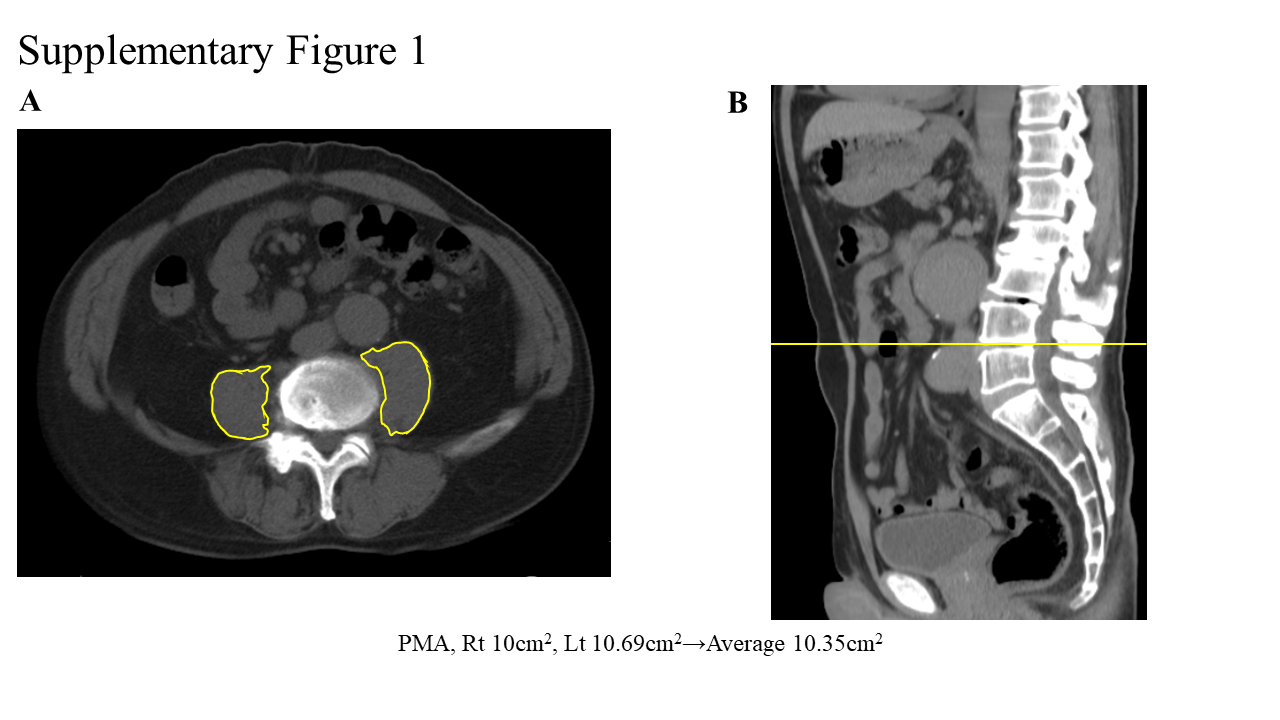

Supplement: S1 Fig — A. axial section B. sagittal section of CT scan. As shown in the yellow line, the bilateral psoas muscles at the level of the fourth lumbar vertebra were manually traced. CT, computed tomography. (TIF) [file pone.0247140.s001.tif]

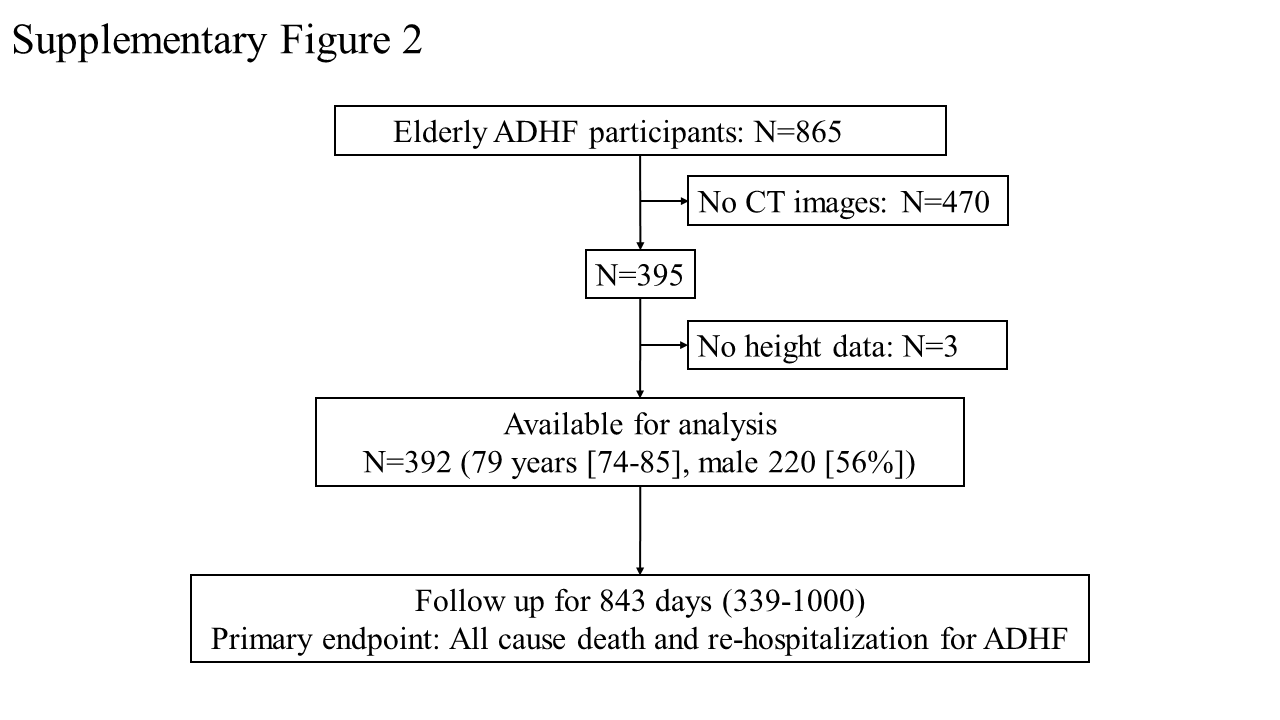

Supplement: S2 Fig — The patients who were hospitalized at the Sakakibara Heart Institute between November 2011 and December 2015 were enrolled. Those who did not undergo abdominal CT during or within 1 year before hospitalization and no height data were excluded. ADHF, acute decompensated heart failure; CT, computed tomography. (TIF) [file pone.0247140.s002.tif]

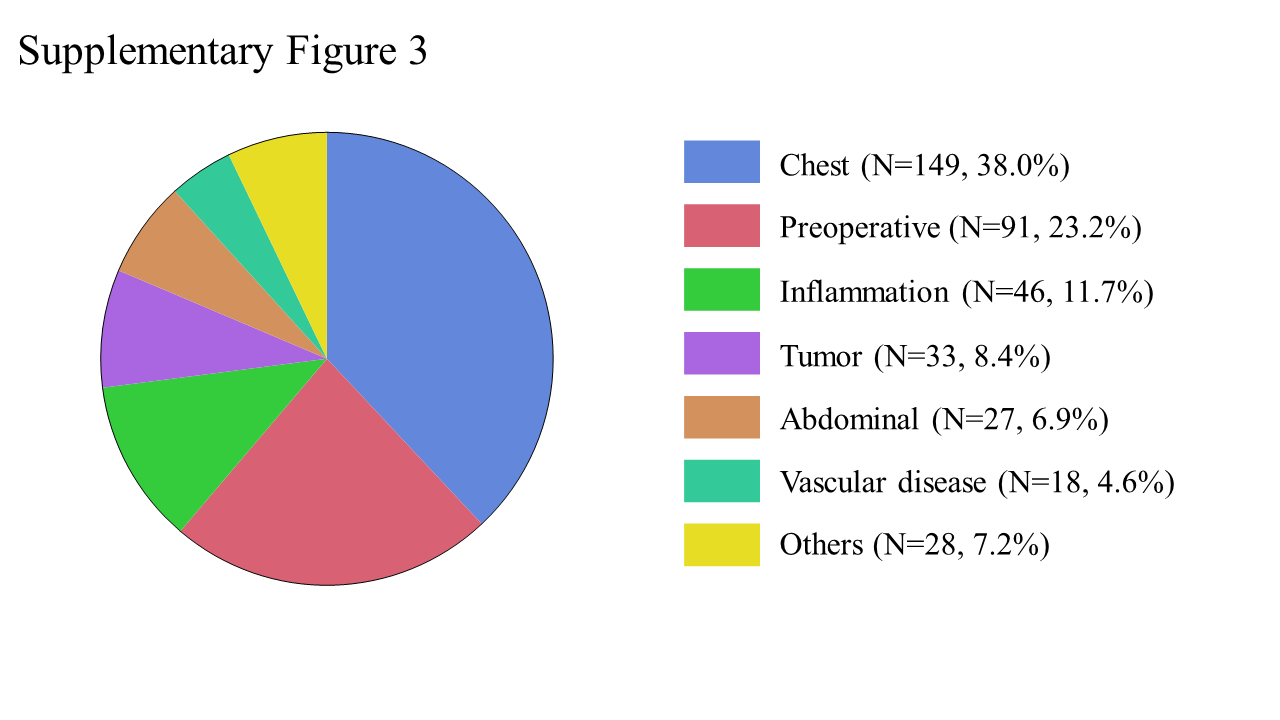

Supplement: S3 Fig — The regions or diseases of interest are shown. The most common reason was evaluation of lung field. CT, computed tomography. (TIF) [file pone.0247140.s003.tif]

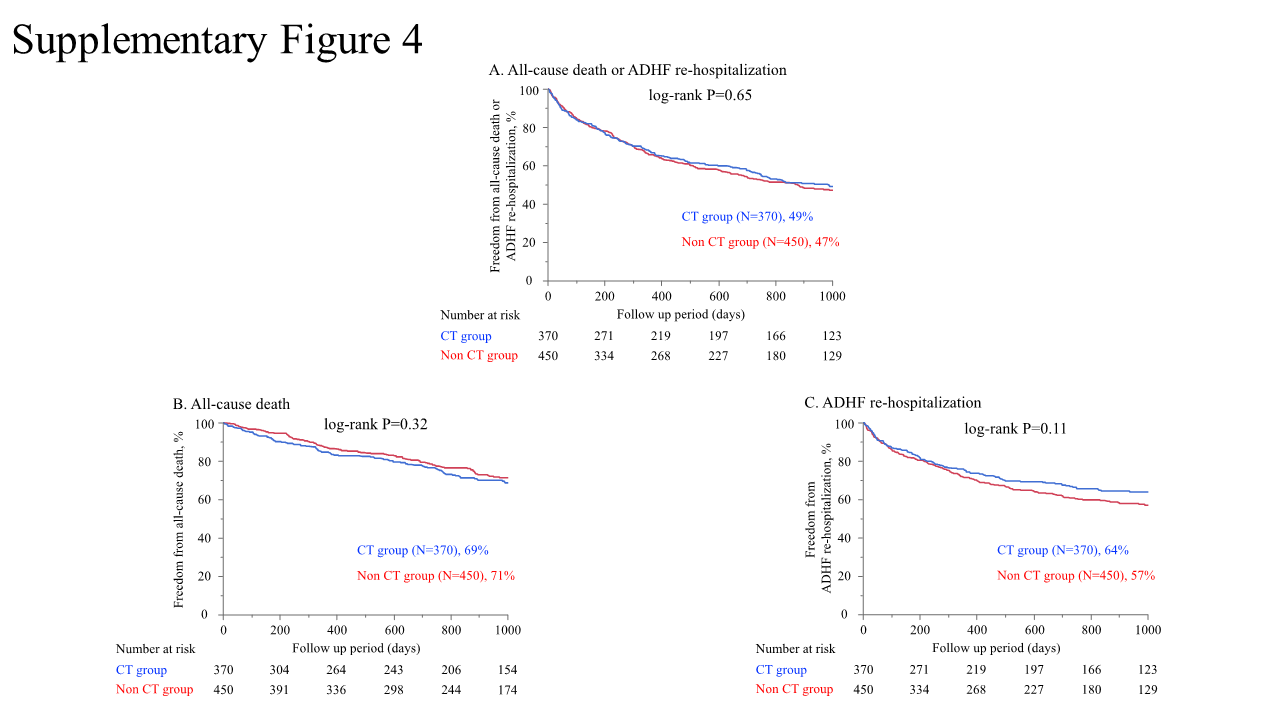

Supplement: S4 Fig — The endpoint was defined as the composite of all-cause death and ADHF re-hospitalization (A.), all-cause death (B.) and ADHF re-hospitalization (C.). ADHF, acute decompensated heart failure; CT, computed tomography. (TIF) [file pone.0247140.s004.tif]
