# Supplementary material for: Low muscle mass assessed by psoas muscle area is associated with clinical adverse events in elderly patients with heart failure
Source: PLoS One. 2021 Feb 16;16(2):e0247140. doi: 10.1371/journal.pone.0247140 (PMC7886171; doi:10.1371/journal.pone.0247140)
Supplement: S1 Table — (PDF) [file pone.0247140.s005.pdf]

S1 Table. Baseline characteristics of CT group and non-CT group

	Overall	CT group (n=395)	Non CT group (n=470)	P value
Age, years	80 (74-85)	79 (74-85)	80 (74-86)	0.14
Male, n (%)	494 (57.1)	222 (56.2)	272 (57.9)	0.62
BMI, (kg/m^2)	22.9 (20.5-25.5)	22.7 (20.2-25.4)	23.0 (20.8-25.5)	0.25
BSA (m^2)	1.57±0.20	1.57±0.20	1.57±0.20	0.83
NYHA, (I/II/III/IV) (%)	0/245/260/358 (0/28/30/42)	0/108/131/156 (0/27/33/40)	0/137/129/202 (0/29/28/43)	0.20
Etiology				<0.001
IHD	268 (31)	116 (29)	152 (32)	
DCM	69 (8.0)	31 (7.9)	38 (8.1)	
VHD	315 (36)	174 (44)	141 (30)	
Past history and comorbidities				
Smoking history, n (%)	414 (48)	193 (49)	221 (47)	0.64
Previous HF hospitalization, n (%)	330 (38)	144 (37)	286 (40)	0.35
Atrial fibrillation, n (%)	505 (58)	216 (55)	289 (61)	0.043
Hypertension, n (%)	582 (67)	255 (65)	327 (70)	0.12
Diabetes mellitus, n (%)	280 (32)	131 (33)	149 (32)	0.65
Dyslipidemia, n (%)	382 (44)	171 (43)	211 (45)	0.62
Previous stroke, TIA, n (%)	131 (15)	63 (16)	68 (15)	0.54
COPD, n (%)	25 (2.9)	12 (3.1)	13 (2.8)	0.81
Hemodialysis, n (%)	8 (0.9)	4 (1.0)	4 (0.9)	1.00
Internal pacemaker, n (%)	91 (11)	36 (9.1)	55 (12)	0.22
ICD, n (%)	45 (5.2)	21 (5.3)	24 (5.1)	0.89
Vital signs				
SBP, mmHg	138 (120-158)	136 (119-153)	140 (120-161)	0.055
DBP, mmHg	77 (65-92)	75 (64-90)	78 (66-95)	0.012
Heart rate, (bpm)	86 (70-108)	86 (70-110)	86 (70-106)	0.38

Laboratory data				
Hemoglobin, (g/dl)	11.6±2.0	11.4±2.0	11.8±1.9	0.001
Serum albumin, (mg/dl)	3.7 (3.4-3.9)	3.7 (3.3-3.9)	3.7 (3.4-3.9)	0.17
eGFR, (ml/min/1.73 m^2)	48 (35-63)	48 (34-63)	48 (36-63)	0.62
NT-proBNP, (pg/μl)	3686 (1913-7903)	4171 (2024-8295)	3405 (1772-7780)	0.049
Echocardiography				
LVEF, (%)	48 (32-59)	50 (34-60)	47 (30-58)	0.074
LVEDD, (mm)	50 (44-58)	51 (44-57)	50 (44-58)	0.54
LVESD, (mm)	37 (30-48)	37 (30-47)	37 (31-49)	0.11
LAD, (mm)	45 (40-51)	45 (40-52)	45 (41-50)	0.92
E/e'	20.3 (14.8 – 29.3)	21.6 (15.3 – 30.9)	19.5 (14.6 – 28.1)	0.020
In-hospital outcome				
Length of hospital stay (days)	14 (10-21)	16 (11-24)	13 (9-17)	<0.001
In-hospital death, n (%)	21 (2.4)	14 (3.5)	7 (1.5)	0.051
Medication at discharge				
Loop diuretics, n (%)	673 (80)	299 (78)	374 (81)	0.41
Thiazide, n (%)	98 (14)	37 (12)	61 (15)	0.28
ACEI/ARB, n (%)	542 (64)	216 (57)	326 (70)	<0.001
β-blocker, n (%)	639 (76)	289 (76)	350 (76)	0.93
MRA, n (%)	280 (33)	123 (32)	157 (34)	0.62
CCB, n (%)	301 (36)	132 (35)	169 (37)	0.58
Statins, n (%)	301 (36)	138 (36)	163 (35)	0.76
Aspirin, n (%)	371 (44)	185 (49)	186 (40)	0.015

BMI, body mass index; BSA, body surface area; NYHA, New York Heart Association, IHD, ischemic heart disease; DCM, dilated cardiomyopathy; VHD; valvular heart disease; HF, heart failure; TIA, transient ischemic attack; COPD, chronic obstructive pulmonary disease; ICD, implantable cardioverter defibrillator; SBP, systolic blood pressure; DBP, diastolic blood pressure; eGFR, estimated glomerular filtration rate; NT-proBNP, N-terminal pro B-type natriuretic peptide; LVEF, left ventricular ejection fraction; LVEDD, left ventricular end-diastolic dimension; LVESD, left ventricular end-systolic dimension; LAD, left atrial dimension; ACEI, angiotensin-converting enzyme inhibitor; ARB, angiotensin receptor blocker; MRA, mineralocorticoid receptor

antagonist; CCB, calcium channel blocker.
